# Opportunities and barriers for prehospital emergency medical services research in the Netherlands; results of a mixed-methods consensus study

**DOI:** 10.1007/s00068-023-02240-w

**Published:** 2023-03-04

**Authors:** Niek J. Vianen, Iscander M. Maissan, Dennis den Hartog, Robert J. Stolker, Robert J. Houmes, Diederik A. M. P. J. Gommers, Nico L. U. Van Meeteren, Sanne E. Hoeks, Esther M. M. Van Lieshout, Michael H. J. Verhofstad, Mark G. Van Vledder, the Dutch Opportunities & Barriers in EMS research group, Jelmer Alsma, David N. Baden, Timo Bevelander, Joost Bierens, Jan Bollen, Jan Bosch, Bert Dercksen, Johannes J. Duvekot, Menno I. Gaakeer, Geert-Jan van Geffen, Dorien H. F. Geurts, Pierre M. van Grunsven, Mark van Heijl, Arnoud van ’t Hof, Markus W. Hollmann, Nico Hoogerwerf, Isabelle C. Huig, Fabian Kooij, Marcel A. de Leeuw, Caroline D. van der Marel, Marlies Morsink, Gerrit J. Noordergraaf, Harm van de Pas, Joost H. Peters, Martijn Poeze, Paul Richelle, Bob Roozenbeek, Patrick Schober, Lisette Schoonhoven, Hans van Schuppen, Arjen Siegers, Dick Tibboel, Rudolf T. Tolsma, Nigel M. Turner, Matthijs de Visser, Lilian C. M. Vloet, Ronald de Vos, Bas van Wageningen, Angelique van der Weerd

**Affiliations:** 1 Trauma Research Unit, Department of Surgery, Erasmus MC, University Medical Center Rotterdam, P.O. BOX 2040, 3000 CA Rotterdam, The Netherlands; 2 Department of Anesthesiology, Erasmus University Medical Center Rotterdam, Rotterdam, The Netherlands; 3 Department of Intensive Care, Erasmus University Medical Center, Rotterdam, Netherlands

**Keywords:** Prehospital, Emergency medicine, Research, Opportunities, Barriers, Emergency medical services

## Abstract

**Introduction:**

Quality improvement in prehospital emergency medical services (EMS) can only be achieved by high-quality research and critical appraisal of current practices. This study examines current opportunities and barriers in EMS research in the Netherlands.

**Methods:**

This mixed-methods consensus study consisted of three phases. The first phase consisted of semi-structured interviews with relevant stakeholders. Thematic analysis of qualitative data derived from these interviews was used to identify main themes, which were subsequently discussed in several online focus groups in the second phase. Output from these discussions was used to shape statements for an online Delphi consensus study among relevant stakeholders in EMS research. Consensus was met if 80% of respondents agreed or disagreed on a particular statement.

**Results:**

Forty-nine stakeholders participated in the study; qualitative thematic analysis of the interviews and focus group discussions identified four main themes: (1) data registration and data sharing, (2) laws and regulations, (3) financial aspects and funding, and (4) organization and culture. Qualitative data from the first two phases of the study were used to construct 33 statements for an online Delphi study. Consensus was reached on 21 (64%) statements. Eleven (52%) of these statements pertained to the storage and use of EMS patient data.

**Conclusion:**

Barriers for prehospital EMS research in the Netherlands include issues regarding the use of patient data, privacy and legislation, funding and research culture in EMS organizations. Opportunities to increase scientific productivity in EMS research include the development of a national strategy for EMS data and the incorporation of EMS topics in research agendas of national medical professional associations.

## Introduction

Over the past decades, prehospital emergency medical services (EMS) in the Netherlands developed tremendously. While ambulance services offered little more than a means of horizontal transportation in the early fifties, ambulances are currently manned by well-trained EMS clinicians able to offer evidence-based prehospital advanced (trauma) life support. Moreover, in severely ill and injured patients, ground EMS by ambulance is complemented by a physician-staffed helicopter emergency medical services (HEMS) [1]. Together with improvements in in-hospital resuscitation and treatment, these developments have led to better outcomes after prehospital cardiac arrest, trauma, and stroke [2, 3].

Progress of EMS would not have been possible without continuous adjustment and gradual improvement of regional and national networks and continuous appraisal and revision of local, regional, and national dispatch criteria and treatment protocols for both ground EMS and HEMS [4–6]. To do so, one would suppose that meticulous quality control and robust data from clinical studies are paramount.

However, the scientific evidence supporting many of the dispatch and treatment algorithms used by both ground emergency medical services and HEMS teams is not always as robust as desired [7, 8]. In addition, available evidence is often derived from in-hospital studies or EMS systems from abroad, which may not apply to the Dutch EMS system regarding level of training, organization of trauma systems, and geographic characteristics. Thus, to gain better understanding of which prehospital interventions do or do not benefit Dutch patients and how prehospital emergency medical and trauma care can be organized in a cost-effective manner, more research tailored to the Dutch situation is needed. And while high-quality EMS research is being undertaken in the Netherlands, there is little national coordination with regard to the organization and execution of EMS research.

Recently, an updated version of the 2016 Dutch EMS research agenda was published by Vloet et al., based on the results of an online Delphi consensus study [9, 10]. Six important themes for future EMS research were identified, most of which focused on organizational aspects of EMS (mostly ground EMS) in the Netherlands. However, an in-depth analysis of current opportunities and barriers for performing research in EMS was not explored. Initiatives from other countries (e.g., Canada) have shown that a focus on opportunities and barriers can be extremely instrumental when it comes to setting priorities for research and improving the basic conditions needed for effective EMS research [11–13]. We hypothesize that this will also be true for the Netherlands. Thus, the objective of this mixed-method approach consensus study was to identify potential opportunities and barriers for research in prehospital emergency care in the Netherlands.

## Methods

This study was designed as a three-phase, mixed-method approach consensus study. Its design was based on the mixed-methods approach used to generate the Canadian national EMS research agenda [14]. Stakeholders currently involved in EMS research were personally invited to participate in the study after being identified using purposive sampling, starting with six Dutch HEMS physicians and ambulance nurses involved in recent prehospital research projects. This list of stakeholders was further expanded using snowball sampling: already identified stakeholders were asked to suggest other stakeholders with relevant activities in EMS research, who were subsequently invited to participate in the study as well. The list of stakeholders was updated throughout the first two phases of the study.

### Phase 1

A semi-structured interview guide was used to steer the (online) interviews (Appendix 1). Participants were invited to elaborate on certain subjects using additional probing questions. Interviews were digitally recorded, transcribed, and stored on a password protected computer. Interviews were held till data saturation was reached. Data saturation was defined as the moment where no new information was gained from at least two interviews. Once the interviews were completed, the qualitative data derived from the interviews were analyzed according to the methodology for thematic analysis as described by Braun and Clarke [14] using software developed specifically for qualitative data analysis (ATLAS.ti version 8, ATLAS.ti, Scientific Software Development GmbH, Berlin, Germany). Topics were discussed and overarching themes were identified. For each theme, several overarching statements—capturing the entire theme—were derived from the list of topics.

### Phase 2

In the second phase, each theme identified in the previous phase was presented in an online focus groups containing two to three participants from previous Phase 1, and at least one new participant. Again, participants were selected using purposive sampling and snowball sampling, based on their input during phase 1 of the study, or if their background was deemed relevant to the specific theme discussed (for instance, a physician with a degree in law and a psychologist with connections to EMS research groups to discuss specific themes). After a short presentation containing background information and results obtained, a discussion of the theme of interest was initiated based on the statements derived from the previous round. Once all focus groups were completed, data were analyzed in a similar way as in the first phase according to the same methodology as mentioned above.

### Phase 3

The third phase of this study consisted of a two-round online Delphi consensus study [15]. For each theme, several statements were generated based on information gathered in the first two phases of the study. These statements were uploaded in Castor EDC, an online electronic data capture system (Castor EDC, Amsterdam, The Netherlands). In the first round, participants were invited to rank the importance of each statement on a five-point Likert scale (1: I don’t agree at all; 5: I fully agree). In addition, participants were asked to provide commentary on each statement. After closure of the first round, statements were adjusted based on the commentary given and statements on which no consensus had been reached in the first round were offered for a second round. For each statement, participants were provided with their own answer from the first round as well as the median answer of all other participants. Participants who completed at least 50% of the first round were asked to rank the remaining statements on a five-point Likert scale during a second online survey round. Answers 1 (I don’t agree at all) and 2 (I don’t agree) were combined and processed as disagreement; similarly, answers 4 (I agree) and 5 (I fully agree) were combined and processed as agreement. When 80% or more of respondents agreed or disagreed on a statement, it was considered as consensus was reached on that particular statement, as is customary for this type of research [14, 15].

### Medical ethics approval

The study protocol was reviewed by the Medical Research Ethics Committee (MREC) of the Erasmus MC, Rotterdam, The Netherlands. The study was waived (MREC nr. Mec-2020-0464) as its content is not covered by the Medical Research Involving Human Subjects Act (Dutch: WMO (Wet Medisch-Wetenschappelijk Onderzoek met mensen)).

## Results

During the first two phases of the study, 60 relevant stakeholders were identified using purposive and snowball sampling. Eventually, 49 stakeholders participated in at least one phase of the study (all were invited but 11 (18.3%) declined to participate for various reasons). Characteristics of participants in all three phases of the study are listed in Table 1.

**Table 1:** Characteristics of participants in each phase of the study

Characteristic	*n* (%)			
	Total	Phase 1	Phase 2	Phase 3
Total number of participants	49 (100%)	22 (100%)	15 (100%)	32 (100%)
Physicians^a^	39 (80%)	19 (86%)	10 (67%)	24 (75%)
Anesthesiologist	15 (31%)	8 (36%)	3 (20%)	12 (38%)
Resident anesthesiology	1 (2%)		1 (7%)	1 (3%)
Trauma surgeon	7 (14%)	2 (9%)	1 (7%)	4 (13%)
ER-physician	6 (12%)	2 (9%)	2 (13%)	4 (13%)
Internal medicine	1 (2%)	1 (5%)		1 (3%)
Pediatrician	1 (2%)	1 (5%)		
Intensive care physician	3 (6%)	1 (5%)	1 (7%)	1 (3%)
Gynecologist	1 (2%)	1 (5%)		
Neurologist	1 (2%)	1 (5%)	1 (7%)	1 (3%)
Cardiologist	1 (2%)	1 (5%)		
General practitioner with interest in emergency care	2 (4%)	1 (5%)	1 (7%)	
Ambulance nurses	5 (10%)	2 (9%)	2 (13%)	5 (16%)
Others	5 (10%)	1 (5%)	3 (20%)	3 (9%)
Manager at the Dutch National Sector Organization for Ambulance Care^b^	1 (2%)	1 (5%)		1 (3%)
Professor emergency care	1 (2%)		1 (7%)	1 (3%)
Professor perioperative care	1 (2%)		1 (7%)	
Researcher on prehospital emergency care	1 (2%)			1 (3%)
Psychologist operating with emergency care personnel	1 (2%)		1 (7%)	
Side tasks				
HEMS physician	11 (22%)	7 (32%)	2 (13%)	5 (16%)
HEMS coordinator	2 (4%)	1 (5%)		2 (6%)
Member of the Scientific Committee of the Dutch National Sector Organization for Ambulance Care^b^	4 (8%)	2 (9%)	2 (13%)	3 (9%)
Medical manager of a regional ambulance care provider	4 (6%)	2 (9%)	1 (7%)	1 (3%)
Legal expert	2 (4%)		1 (7%)	2 (6%)
Member of the Scientifical Advisory Board of the National Network of Emergency Care^c^	2 (4%)	2 (9%)		
Chairman of the Netherlands Emergency department Evaluation Database	1 (2%)		1 (7%)	1 (3%)

^a^Currently active in clinical care

^b^Dutch National Sector Organization for Ambulance Care; AZN (AmbulanceZorg Nederland)

^c^Scientifical Advisory Board of the National Network of Emergency Care; WAR (Wetenschappelijke AdviesRaad) LNAZ (Landelijk Netwerk Acute Zorg)

### Phase 1

In the first phase of this study, a total of twenty-two interviews were held between July 15, 2020, and October 2, 2020. Data saturation was reached after 22 interviews. Thematic analysis of the interviews resulted in five main themes being identified. These themes were: (1) data registration and data sharing, (2) laws and regulations, (3) financial aspects, (4) organization and culture, and (5) incentives for performing research.

### Phase 2

Focus groups per theme took place between February 23, 2021 and March 31, 2021, and were held based on eighteen statements derived from the data gathered in the first step of this study, which are shown in Appendix 2. No new themes were identified, but the themes (4) organization and culture and (5) incentive for performing research showed such a high level of overlap it was agreed to merge these two themes for the remainder of the study.

### Phase 3

The third phase of this study was a two-round online Delphi survey. Fifty-three stakeholders were asked to participate. Information gathered in the first and second phase of this study was subsequently converted into thirty-three statements. Eventually, 32 of 53 stakeholders (60.4%) completed the first survey round and consensus was reached on thirteen statements (39.4%). The twenty statements for which no consensus was reached were send back to the stakeholders. Twenty-seven stakeholders (50.9%) completed the second survey round, and consensus was reached for an additional eight statements, setting the total number of statements with consensus at 21 (63.6%). All statements and their level of consensus are shown in Table 2. A brief overview of opportunities for Dutch EMS research is given in Table 3.

**Table 2:** Statements used for online Delphi consensus study

	Statement	Percent consensus (all participants)	Percent consensus (participants with clinical EMS duties) (*n* = 12)	Percent consensus (participants without clinical EMS duties) (*n* = 20)
Consensus reached in Delphi round 1	Combining different data sets for scientific research and quality control is made more difficult by the multitude of different data registration systems within Dutch prehospital emergency medical care	93	91	93
	Incorrect and/or incomplete registration at the source (ambulance form/HEMS^a^ database) has major consequences for the reliability of results of retrospective medical scientific research in prehospital emergency medicine	91	91	95
	Regional ambulance care providers should make greater use of the expertise available within universities, colleges, and (university) hospitals to make scientific research in prehospital emergency medicine more feasible	90	100	83
	It is currently too difficult to combine patient data from various regional ambulance care providers and hospitals for scientific purposes	89	82	93
	The fact that all health data collected prehospital are digitally stored offers great opportunities for retrospective research	88	82	89
	It is not always clear to both researchers and the Medical Ethical Review Committees^b^ how the regulations surrounding the informed consent procedure can be applied for prospective scientific research in prehospital emergency medicine	87	82	94
	Facilitating scientific research (including funding) should be part of the core tasks of every regional ambulance care provider	84	91	78
	A national cross-disciplinary research agenda for prehospital emergency medicine can be an important impetus for better national collaboration with regard to setting up and conducting research in prehospital acute medicine	84	82	89
	There is little or no uniformity between regional ambulance care providers, HEMS^a^ and hospitals in the registration of data from patients with an emergency care requirement	83	90	75
	Implementation and validation of the results of scientific research in prehospital emergency medicine is currently insufficiently done	83	82	80
	The low user-friendliness and limited automation of data registration systems used by the Dutch regional ambulance care providers and HEMS^a^ teams leads to incorrect or incomplete registration of patient data	82	88	83
	Scientists/researchers in prehospital emergency medicine are insufficiently aware of the possibilities for obtaining funding for scientific research	81	64	92
	The implementation of a basic ambulance care data set is an important first step toward national uniform data registration	80	64	88
Consensus reached in Delphi round 2	Patient data in databases of regional ambulance care providers are currently insufficiently accessible for scientific research in prehospital emergency medicine	92	100	85
	The importance of adequate registration of patient data for scientific research is currently insufficiently addressed in ambulance nurse training	89	90	88
	The Medical Ethical Review Committees^b^ in the Netherlands are insufficiently aware of the circumstances under which scientific research is conducted in prehospital emergency medicine	87	91	83
	There is currently insufficient funding available to set up and conduct scientific research in prehospital emergency medicine	87	80	92
	Research in prehospital emergency medicine should have a higher place on the research agenda of the Dutch medical professional associations (or sub-associations if more appropriate) involved	85	82	88
	There is uncertainty about the application of the General Data Protection Regulation^c^ with regard to the use and sharing of health data from patients with emergency care needs for scientific research	81	73	87
	Smart ICT solutions (e.g., automated and anonymous retrieval of decentralized data, only data required for specific research questions) are preferred over central data storage with regard to health data of patients with an emergency care requirement	81	70	88
	In order to achieve innovation and quality improvement, substantial investments must be made in a better research culture within the Dutch ambulance care providers and HEMS teams	81	91	73
No consensus reached	Healthcare institutions involved with prehospital emergency medical care (regional ambulance care providers and HEMS^a^ teams) must invest more in a basic research structure and preconditions for setting up and conducting scientific research	78	100	63
	A professor of prehospital emergency medical care must be appointed in The Netherlands	77	90	69
	Insufficient attention is paid to the importance of prehospital medical scientific research in ambulance personnel training	74	70	78
	Collecting, storing, and making available prehospital patient data for scientific research and quality control requires a national approach	73	73	73
	The current legislation and regulation regarding drug research in patients is not suitable for conducting drug research in prehospital emergency medical care, for example, regarding the issuance and storage conditions of study medication	71	67	75
	With a high-quality research proposal, there are plenty of opportunities to obtain funding for prehospital emergency medical care research	67	50	79
	The existing prehospital emergency medical care network in the Netherlands (National Network of Emergency Care^d^, Regional Consultation Emergency Care Chain^e^, regional ambulance care providers) offers possibilities for regional and/or national coordination of scientific research in prehospital emergency care	64	55	71
	There must be a national quality registration for prehospital emergency care medicine (provided by regional ambulance care providers and HEMS^a^ teams)	63	50	71
	The research agenda for the Dutch ambulance sector drawn up by the HAN University of Applied Sciences^f^ on behalf of the Dutch National Sector Organization for Ambulance Care^g^ is an important impetus for the national organization of scientific research in prehospital emergency care	57	56	58
	The Dutch National Sector Organization for Ambulance Care^g^ must play an important facilitating role in setting up, implementing and applying for funding for scientific research in Dutch ambulance care	52	55	50
	The National Network of Emergency Care^d^ must play an important coordinating role in drawing up a knowledge agenda for prehospital emergency medical care in The Netherlands	52	45	57
	Current Dutch and European legislation and regulations offer sufficient opportunities for conducting high-quality scientific research in prehospital emergency care	44	40	47

^a^MREC; METC (Medisch Ethische Toetsings Commissie)

^b^HEMS; Helicopter Emergency Medical Services

^c^GDPR; AVG (Algemene verordening gegevensbescherming)

^d^National Network of Emergency Care; LNAZ (Landelijk Netwerk Acute Zorg)

^e^Regional Consultation Emergency Care Chain; ROAZ (Regionaal Overleg Acute Zorgketen)

^f^HAN University of Applied Sciences; HAN (Hogeschool van Arnhem en Nijmegen)

^g^Dutch National Sector Organization for Ambulance Care; AZN (AmbulanceZorg Nederland)

**Table 3:** Opportunities for Dutch EMS research identified in this study

Theme	Opportunity
Data registration and data sharing	Develop a national data strategy for EMS research
	Increase reliability and completeness of data by technological innovations
Laws and regulations	Increase familiarity with laws and regulations regarding EMS research among MREC, EMS providers, and EMS researchers
	Publish a good clinical practice guideline for data sharing in EMS research with regard to GDPR regulations
Funding	Intensify collaboration between EMS providers and university medical centers, form research consortia
	Adopt EMS related topics in research agendas of Dutch medical professional associations
Culture and organization	Incorporate teaching about research in training of ground EMS providers
	Emphasize how research findings are adopted in national guidelines

### Data registration and data sharing

Registration, storage, and sharing of patient data proved to be one of the most important themes both enabling and hampering effective EMS research; 11 out of 21 statements on which consensus was reached in the Delphi study pertained to this theme. Barriers for EMS research mentioned during the interviews and focus groups and confirmed in the Delphi rounds included the absence of a common data set for emergency patients among different providers (HEMS, ground EMS, hospital emergency department), precluding effective exchange and merging of these data. The multitude of different electronic patient files used by different services further aggravates this problem. As one of the participants stated during an interview: “At this moment, the ambulance data registration system does not communicate well with the HEMS data registration system, neither with the hospital data registration system. So there are three systems for one patient in the first hour of its care and those systems don’t talk to each other.” Initiatives such as “the minimal data set for ground EMS” and the Dutch Emergency Medicine Database (NEED) were generally considered to be important steps toward achieving this goal. Moreover, the fact that all patient data in the Netherlands is already electronically recorded and stored was considered to be an important condition for such developments by most participants.

In addition, several participants had concerns regarding the quality of routinely gathered prehospital patient data. Currently, correct registration of health data at the source is hampered by limited compliance of prehospital personnel and poor ergonomics of data capture systems, resulting in incomplete and sometimes unreliable data. While non-compliance could be addressed during training of EMS workers as mentioned by some participants, improving ergonomics and data export for data capture requires more technical solutions.

### Laws and regulations

Many participants commented on Dutch and European laws and regulations pertaining to EMS research. Two important subthemes within this theme were (1) informed consent in EMS research and (2) correct application of the European General Data Protection Regulation (GDPR) in EMS research. With regard to informed consent procedures, several participants commented that while there are laws in place that allow inclusion of emergency patients in prospective studies by so-called deferred consent, many prehospital healthcare workers and even members of MREC’s are unfamiliar with this kind of consent procedures. Moreover, the majority of participants felt that not all MREC’s are aware of the intricacies of prehospital EMS research, often leading to a delay in the review process *(*“MREC’s have no idea what EMS-work entails”).

Variable interpretation among different services of the GDPR was considered to be another threat for effective EMS research. As one participant stated; “There are many parties that are afraid of making a mistake (with regard to GDPR, red.).” As stated during one of the focus groups, there is considerable variation between organizations involved in emergency care with regard to their practices in data sharing. Having a uniform national policy based on best GDPR practice for emergency care research data exchange between emergency care organizations including EMS providers was considered to be a great opportunity in the same focus group.

### Financial aspects

Opinions with regard to funding opportunities were widespread during the interviews. Some experienced a total inability to acquire funds for EMS research. As one of the interviewees states “the amount of work to submit a grant proposal is so disproportionally large compared to the chance of actually obtaining the grant, I don’t even get started anymore.” On the other hand, several other interviewees mentioned examples of successfully funded EMS research projects; these were most often projects initiated by researchers from university medical centers (medical specialists) collaborating with EMS organizations for their studies. In the Delphi consensus study, there was agreement on several statements regarding funding opportunities for EMS research. These were considered to be generally limited for EMS research. Participants in the study also agreed that part of this may be due to limited knowledge of funding opportunities among EMS workers and that this may be improved by further collaboration between EMS organizations and research institutes like university medical centers. In addition, during the focus group on this theme, the importance of national collaboration in research consortia for acquiring funds was mentioned as an important opportunity by several participants. At last, medical professional associations may play an important role in increasing the chance of funding being allocated for EMS projects, by including knowledge gaps regarding prehospital treatment of conditions of interest in their respective research and knowledge agendas.

Another important financial barrier for EMS research mentioned in the interviews was the limited amount of resources available to those who want to be involved in EMS research. As stated, some larger EMS providers have indeed allocated funds and time to accommodate EMS research activities, but many others do not. As one participant stated; “When you want to acquire funding for research, you first have to get the basics right.” In order to get these basics right, participants agreed that facilitating research should be among the core tasks of ambulance service providers and HEMS services. To achieve this, EMS organizations should allocate internal funds to provide basic conditions for research activities, such as allocated time, adequate (IT) facilities, and opportunities for learning about doing research.

### Organization and culture

The theme “organization and culture in EMS research” was widely elaborated on by participants. There was general agreement that substantial investments should be made to achieve a better research culture among EMS organizations. Examples from the interviews of current barriers include little incentive for doing research among EMS organizations, lack of enthusiasm for and knowledge about research among EMS workers (“if you sign up for ambulance work, you don’t sign up for research”), and little perceived benefits for the patient due to a lack of feedback from past research projects. Indeed, implementation of scientific results from EMS research in treatment protocols was perceived to be insufficient at the moment. Several participants praised the foundation of the scientific committee with the Dutch National Sector Organization for Ambulance Care (Dutch: AmbulanceZorg Nederland) and the publication of the national research agenda for (ground) EMS for their contribution to establishing a vital research culture among EMS organizations. However, no consensus was met on statements involving these initiatives in the Delphi rounds, and many participants mentioned that such initiatives can only be successful if they aim for intensive collaboration between ambulance services, HEMS services, and research institutes on a national level. Emphasizing how research may change daily practice by quickly implementing findings from national studies in national guidelines could further contribute to a viable research culture.

## Discussion

In this mixed-methods consensus study, opportunities and barriers for performing research in EMS in the Netherlands were examined. Four themes important for EMS research in the Netherlands; were identified: (1) data registration and sharing, (2) laws and regulations, (3) financial aspects, and (4) organization and culture.

Probably the most challenging topic identified in this study is the way health data are currently collected, stored, and shared for EMS research in the Netherlands. As vast amounts of health data are currently collected for each patient traveling through the emergency care system, improvements in data capture and handling have a huge potential regarding quality control and research [16]. Unfortunately, as this study shows, tapping into this potential is currently hindered by several factors. First, manual entry of patient characteristics and vital parameters in EMS electronic medical records creates a risk of incomplete and unreliable data being stored. Second, various data sets used by various EMS providers combined with a myriad of software applications unable to communicate with each other and incapable of running simple (or complex) data queries make combining the data sets from HEMS, ground EMS, and hospitals almost impossible. Thirdly, varying interpretation of privacy laws and regulations with regard to data sharing between organizations (leading to data sharing practices ranging from liberal sharing of data to outright refusal to share any data) leads to difficult and time consuming procedures when data sets need to be acquired and combined. Research projects that have succeeded in combining data from different providers often had to come up with complicated legal constructs [17].

The current study provides several suggestions to address these issues. First, arrangements should be made to align the way data are currently being collected among different organizations involved in the emergency treatment of critically ill or injured patients. Data sets should be easy to combine, contain the same variables, should be easy to query for scientific purposes and quality control, and should abide to the FAIR principles [18], all with respect for patients’ privacy and relevant laws and regulations. National data registries such as the National Trauma Registration (Dutch: Landelijke TraumaRegistratie; LTR) and Netherlands Intensive Care Evaluation (NICE) and examples such as the Danish database for prehospital emergency medical services could serve as examples to build upon [19–21]. Second, knowledge regarding (privacy) rules and regulations should be increased among all organizations involved, by education and—for example—publication of a best practice guideline for EMS research and privacy (and other) regulations. Finally, the reliability of data can be improved by increasing the level of automation and ergonomics of data registration systems. Automated registration and storage of vital parameters, speech capture software, and automatic linking of the registered data to a unique personal patient number will all facilitate the right information being accurately and completely recorded and linked to the right patient.

Participants in our study stated that investing in a research culture among EMS workers may have a positive impact on EMS research in the Netherlands. Incorporating research activities in training as well as emphasizing the importance of research and making the impact of research on day-to-day clinical work more visible (e.g., development of decision aids) may further increase awareness and enthusiasm. Ambulance nurses and HEMS physicians interested in performing or participating in research projects should be encouraged to do so. This can be done by offering them time, funds, and support to develop their own research ideas and should been given tools to bring these interests to practice successfully. EMS research agenda’s from other countries have identified similar issues; lack of time and resources was identified as one of the major threats to a vital research culture in the Canadian EMS research agenda too [11]. In addition, EMS workers with less research ambition can be involved by emphasizing the importance of evidence-based practice and by organizing journal clubs and research presentations to offer a clear outlook on how (national) research can lead to better patient care and new guidelines. In order to make these changes happen, it is important that individual ambulance care and HEMS organizations expand their focus from pure operational aspects toward a more quality-of-care-driven focus. While not all ambulance services may be able to initiate research on their own, all ambulance and HEMS services in the Netherlands should be able to accommodate research projects initiated by other parties, preferably based on a national policies. At last, by intensifying cooperation between ambulance services and (university) medical centers on a regional and national level, resources and experience can be combined and new projects can be more successful. The fact that most studies in Dutch EMS research have been initiated by (university) medical centers, shows that there is great interest in this particular field of care in academia, which could be improved by more systematic collaboration between hospitals and EMS providers.

In terms of funding, participants perceived that funding for EMS research is scarce and that many involved in EMS research are not informed about existing funding opportunities. Improvement of the aforementioned collaboration between ambulance services and (university) medical centers may increase knowledge to funding opportunities for EMS researchers. Likewise, participants stated that scientific organizations (such as the Dutch associations and societies for trauma surgery, internal medicine, cardiology, anesthesiology, neurology, and emergency medicine) could have an important role in expanding opportunities for EMS research funding by incorporating prehospital treatment of ailments within their specific area of interest on their research agendas.

In the 2015 Canadian Research Agenda for EMS research, most strengths, opportunities, barriers, and recommendations pertained to funding, time for doing research, and organizational culture [12]. Likewise, the 2003 USA research agenda addresses five major impediments for the performance of high-quality EMS research in the USA. Four of these major impediments are similar to our findings and address funding, data organization and collection, informed consent, and research culture [13]. The Dutch EMS system differs markedly from EMS systems in Canada and the USA (ambulance nurses instead of EMS technicians, national coverage of physician-staffed HEMS, geographic differences), and significant time has passed since the Canadian and USA research agendas were published. However, similarities between the outcomes of aforementioned research agendas and the current study suggest that challenges in performing prehospital EMS research are universal and not easily to overcome. Identifying barriers and formulating opportunities is therefore an important first step to increase scientific productivity in a given prehospital system and it may be advocated that similar studies like this study are performed in other countries as well [16, 22, 23].

Several limitations apply to this study. First, the overrepresentation of physicians in all phases of this study may have colored the overall results. However, the majority of EMS research in the Netherlands is initiated and conducted by physicians and many of the physicians participating in this study have connections to ambulance services. We therefore feel our sample to be representative of the EMS research landscape in the Netherlands as it currently is. In addition, for our focus groups, we were limited to small groups and relatively short sessions due to COVID restrictions at the time of the study. Ideally, a moderated session involving all stakeholders at the same time would have been preferred, as this would have given all participants the opportunity to extensively reflect on each identified theme. While this may have impacted on the development of the statements for the Delphi study, all statements were subjected to multiple rounds of consensus formation in the subsequent Delphi study, leveling out the effect of the small focus groups.

## Conclusion

Barriers for prehospital EMS research in the Netherlands include issues regarding the use of patient data, privacy and legislation, funding, and research culture in EMS organizations. Opportunities to increase scientific productivity in EMS research include the development of a national strategy for EMS data and the incorporation of EMS topics in research agendas of national medical professional associations.

## Data Availability

Data will be made available upon reasonable request to the corresponding author.

## Appendix 1: Semi-structured interview guide

1. In what way are you involved in prehospital emergency medical care (research) in the Netherlands?
2. In what type of research are you (currently) involved?
3. In which area of prehospital emergency medical care are you conducting research?
4. What facilitates prehospital emergency medical care research in the Netherlands?
5. What are the opportunities for prehospital research in the Netherlands?
6. What barriers did you encounter conducting prehospital emergency medical care research in the Netherlands?
7. What are your thoughts about the way prehospital emergency medical care research is currently funded in the Netherlands?
8. Do the results of (published) studies find their way to the prehospital emergency medical care protocols in the Netherlands?
9. Is it of importance that the opportunities and barriers for prehospital emergency medical care research in the Netherlands are identified to carry out future research?
10. What are three areas within prehospital emergency medical care that need more research?
11. Have any studies been carried out abroad that you think should be repeated/re-examined in the Netherlands?
12. Are there any relevant topics within prehospital emergency medical care that we have not discussed yet?
13. Who else do you think should be interviewed regarding this topic?

## Appendix 2: Statements used for focus groups

**Table:**

Theme	Statement
Data registration and data sharing	All prehospital collected patient data should be stored in a central database
	Thorough quality control at a national level is impossible without uniform data collection and storage
	The Dutch/European legislation and regulations with regard to the storage of patient data is a major obstacle in organizing a national data strategy
	Due to the unreliability of the entered data, all initiatives to store and make prehospital patient data available on a large scale are pointless in advance
	Research in prehospital emergency care in the Netherlands would benefit from more collaboration
Laws and regulations	The General Data Protection Regulation (GDPR) and its variable interpretation are a major obstacle to conducting research in prehospital emergency care
	Separate legislation and regulations must be drawn up for medical scientific research outside the hospital
	A designated Medical Ethics Review Committee (MREC) with knowledge about the assessment of prehospital medical scientific research should be installed
Financial aspects	There are currently insufficient funding options for conducting research in prehospital emergency care in the Netherlands
	There should be a national funding program for scientific research in prehospital emergency care in the Netherlands
	Regional ambulance care providers should be obliged to invest part of their turnover in scientific research
	The Dutch National Sector Organization for Ambulance Care should have a facilitating role in financing research projects in prehospital emergency care
Organization and culture	The Dutch prehospital emergency care has a long and thorough research history
	Quality control and innovation through scientific research should be among the core tasks of every ambulance care provider
	Every ambulance worker in the Netherlands should be trained in academic skills
Incentive for performing research	The Dutch prehospital emergency care is in need of a professor of prehospital emergency care
	There should be a financial incentive for conducting research in ambulance care
	There should be a mandatory quality control for ambulance care
